# Stillbirth in women with Type 1 Diabetes mellitus—still a current topic

**DOI:** 10.1007/s00404-024-07609-9

**Published:** 2024-07-03

**Authors:** Susanne Dargel, Jana Westphal, Christof Kloos, Ekkehard Schleußner, Friederike Weschenfelder, Tanja Groten

**Affiliations:** 1 Department of Obstetrics, Jena University Hospital, Am Klinikum 1, 07747 Jena, Germany; 2 Department for Internal Medicine III, Jena University Hospital, Jena, Germany

**Keywords:** Intrauterine death, Diabetes mellitus type 1, Diabetes in pregnancy

## Abstract

**Purpose:**

Compared to the general stillbirth rate in Germany for term deliveries of 0.12% the risk in type 1 diabetes mellitus is reported to be up to ten times higher. The reasons for this excess risk of intrauterine demise are still not fully elucidated. Risk factors named in the literature include poor glycemic control before and during pregnancy and the occurrence of ketoacidosis. Additionally there might be a diabetes related type of placental dysfunction leading to organ failure in late pregnancy. Understanding the underlying causes is mandatory to develop strategies to reduce the incidences. The Purpose of this publication is to point out the difficulties in prediction of intrauterine death in pregnant type 1 diabetes patients and thus emphasizing the necessity of constant awareness to all caregivers.

**Methods:**

We present a case series of four cases of stillbirth that occurred in patients with type 1 diabetes mellitus at our tertiary care obstetric unit during a five-year period.

**Results:**

In all four presented cases the underlying cause of intrauterine demise was different and we could not find a common mechanism or risk profile. Furthermore, established monitoring tools did not become peculiar to raise awareness. We compared our cases to published data. Underlying causes of intrauterine death in type 1 diabetes are discussed in the light of the current literature.

**Conclusions:**

The main risk factors of stillbirth in diabetic pregnancies are high maternal blood glucose levels including pre-conceptional HbA1c and diabetic ketoacidosis. Late acute placental insufficiency are associated with intrauterine death in type 1 diabetes. Despite the elevated risk of near term intrauterine demise there are currently no guidelines on how to monitor pregnancies in type 1 diabetes for fetal distress during the third trimester. Established thresholds for fetal Doppler data indicating fetal distress in normal and growth restricted fetuses may not be applicable for overgrown fetuses. Future research on how to monitor the diabetic fetus needs to be initiated.

## What does this study add to the clinical work

**Table:** 

Stillbirth in type 1 diabetes mellitus is still a topic. Since most cases are unpredictable caregivers need to sustain constant awareness of the elevated risk in the presence and the absence of common risk factors.	

## Background

Despite all the medical progress that has led to a significant improvement in obstetrical outcome all over the world, women with preexisting diabetes are still at high risk for severe complications during pregnancy including stillbirth [1]. The term stillbirth generally applies to a baby born with no signs of life and includes the birth of a baby following fetal death before onset of labor (antepartum stillbirth) or during labor or birth (intrapartum stillbirth). Hug and authors published an updated review of worldwide stillbirth rates in 2021 and reported a global rate of 13.9 stillbirth per 1000 total birth at 28 weeks or more with a rate of 2.9 for Europe. The worldwide stillbirth rate dropped between 2000 and 2019 from 21.4 to 13.9 by 35.1% and in Western Europe from 3.9 to 2.9 by 25.7%.

While the rate of stillbirth in the general obstetrics population has been decreasing over the last decades, pregnant women with a preexisting diabetes still have a 4-to fivefold increased risk for stillbirth. Fretts reported in 1992 changes in cause-specific fetal death rates in a population of a tertiary care hospital unit. The fetal death rate (per 1000 births) among 88,651 reported births decreased from 11.5% in the 1960s to 5.1% in the 1980s [2]. Remarkably, the risk for fetal death in pregnancies complicated by hypertension was significantly elevated in the 1960s with 2.39 [2.13–2.68] and dropped to 1.54 [0.94–2.53] in the 1980s, no longer constituting a significant risk. In contrast rates of fetal death in women with insulin treated diabetes were 26 per 1000 births accounting for an RR of 4.24 [2.11–8.53] and dropped to 7.4 (per 1000 births) still accounting for a significant increased RR of 3.27 [1.04–10.3] [2].

In a population-based study Persson reported in 2009 data of 5,089 type 1 diabetic pregnancies and 1,260,207 control pregnancies in Sweden. They report a stillbirth rate of 1.5% and a perinatal mortality rate of 2% in type 1 diabetes compared to 0.3% and 0.48% in the non-diabetic population corresponding to OR of 3.34 [2.46–4.55] for stillbirth and 3.29 [2.50–4.33] for perinatal mortality [3]. The results were adjusted for group differences in maternal age, parity, BMI, chronic hypertensive disease, smoking habits, and ethnicity. More recent studies from Europe and the US reporting pregnancy outcome of cohorts of type 1 diabetes, still describe stillbirth rates between 1 and 2%. [4–11] Compared to the general stillbirth rate in Germany for term deliveries of 0.12% the risk in type 1 DM seems to be even ten times higher (Bundesauswertung nach QSKH-RL 2020 (iqtig.org)). This alarming excess in risk for stillbirth in pregnancies with preexisting type 1 diabetes is still not fully understood and the reasons for intrauterine demise still need to be elucidated. Understanding the underlying causes is mandatory to develop strategies to reduce the incidences.

To further evaluate specific circumstances causing intrauterine death in women with type 1 diabetes, we present four cases of intrauterine death presented in our tertiary care obstetric unit during a five-year period and put them in context with the previously published literature.

## Methods

From 2015 to 2020, 131 patients with type 1 diabetes delivered at our hospital. Four cases of intrauterine death occurred (0.3%). Our hospital offers outpatient care for type 1 diabetic patients in an interdisciplinary team including obstetricians, diabetologists, midwifes and diabetes consultants. Thus, most patients delivering in our obstetric unit have been regularly consulted in our outpatient department. During routine examinations, metabolic parameters, including HbA1c, were collected and ultrasonography with biometry and Doppler examination was performed according to DEGUM standards. Data on medical history, the course of pregnancy and circumstances of intrauterine death are reported for the four observed cases. Fetal weight was determined after birth and placental weight was derived from the histopathologic reports. (Table 1) Ethical approval has been obtained by the ethical committee of the Friedrich-Schiller University in Jena (2022-2672-Daten). The anonymous use of clinical data for research and educational purposes is covered by the governmental rules of Thuringia. Researchers and clinicians are allowed by law to use patient data obtained during routine care to present in scientific and educational reports and analysis.

**Table 1:** Comparative presentation of four cases of intrauterine death in type 1 diabetes pregnancies

	Case 1	Case 2	Case 3	Case 4
Maternal age	23 y	19 y	22 y	40 y
Gravida/Para	IIG, 0P	IG	IG	IIIG, IIP
BMI (kg/qm)	20	22	19	43
Time point of diagnosis of type 1 diabetes (age years)	15	2	During pregnancy	30
Pre-existing conditions other than diabetes	None	None	None	Hypertension
HbA1c at diagnosis of pregnancy	6.0%	10.5%	13,5%	6.5%
Planned pregnancy	YES	NO	NO	NO
Therapy compliance	NO	NO	NO	NO
Disease acceptance	YES	NO	NO	YES
Last ultrasound before diagnosis of IUFT^a^	24	35 + 1	n.a	36
Pulsatility index umbilical artery (PI AU)	n.d	1,09	n.a	1,10
Pulsatility index arteria cerebri media (PI ACM)	n.d	1,57	n.a	1,43
CPR (PI ACM/PI AU) (pctl)	n.d	1,44 (2)	n.a	1,10 (1)
DKA^b^	Yes, euglycaemic	No	No	No
Fetal weight g (pctl)	1040 g (59)	3220 g (70)	2080 g (> 95)	4450 g ( 97)
Placental weight g (pctl)*	343g (50– 90)	305g (< 3)	420g (50)	199g (< < 3)
Birthweight/placental weight ratio (pctl)*	3.03 (50)	10.56 (> > 97)	4.95 (50–90)	22.36 (> > 97)

^a^Intraueterine death, ^b^diabetic ketoacidosis, *according to [12]

## Case presentations

### 1st Case

At 27 weeks of gestation, a 23-year-old white gravida 2 para 0, with a type 1 diabetes (first diagnosis 8 years ago, HbA1c periconceptual 6%) on intensified insulin regimen was transferred to our hospital from a peripheral clinic because of persisting hematemesis, presenting in a desolate condition. No cardiotocographic control of the fetus had been performed and insulin delivery was stopped by the external medical care providers, reasoned by starvation of the patient for more than 24 h, as documented in the patient’s records. After reaching our labor ward, only an untypical flickering of the fetal heart could be visualized while the fetal heart frequency was about 110bpm. The patient presented with ketonuria and glucosuria while being in euglycemic (maternal blood glucose 8 mmol/l). With the arrival at our ward we immediately started insulin infusion therapy as well as excessive rehydration with normal saline. 20 min later, no fetal heart action could be detected anymore. In that critical, unstable maternal metabolic situation no emergency c-section was performed, so fetal loss during an euglycemic diabetic ketoacidosis was diagnosed. Two hours after starting the initial therapy, blood gas analysis still showed a severe metabolic acidosis of the mother: pH 7,25, BE -16 mmol/l, HCO3- 9,6 mmol/l, pCO2 2,97 kPa Glc 13,2 mmol/l. After stabilizing the patient condition, induction of labor was started with misoprostol. 24 h later, our patient gave birth to a macerated girl with a birth weight of 1040 g (59% percentile).

### 2nd Case

**Fig. 1:**
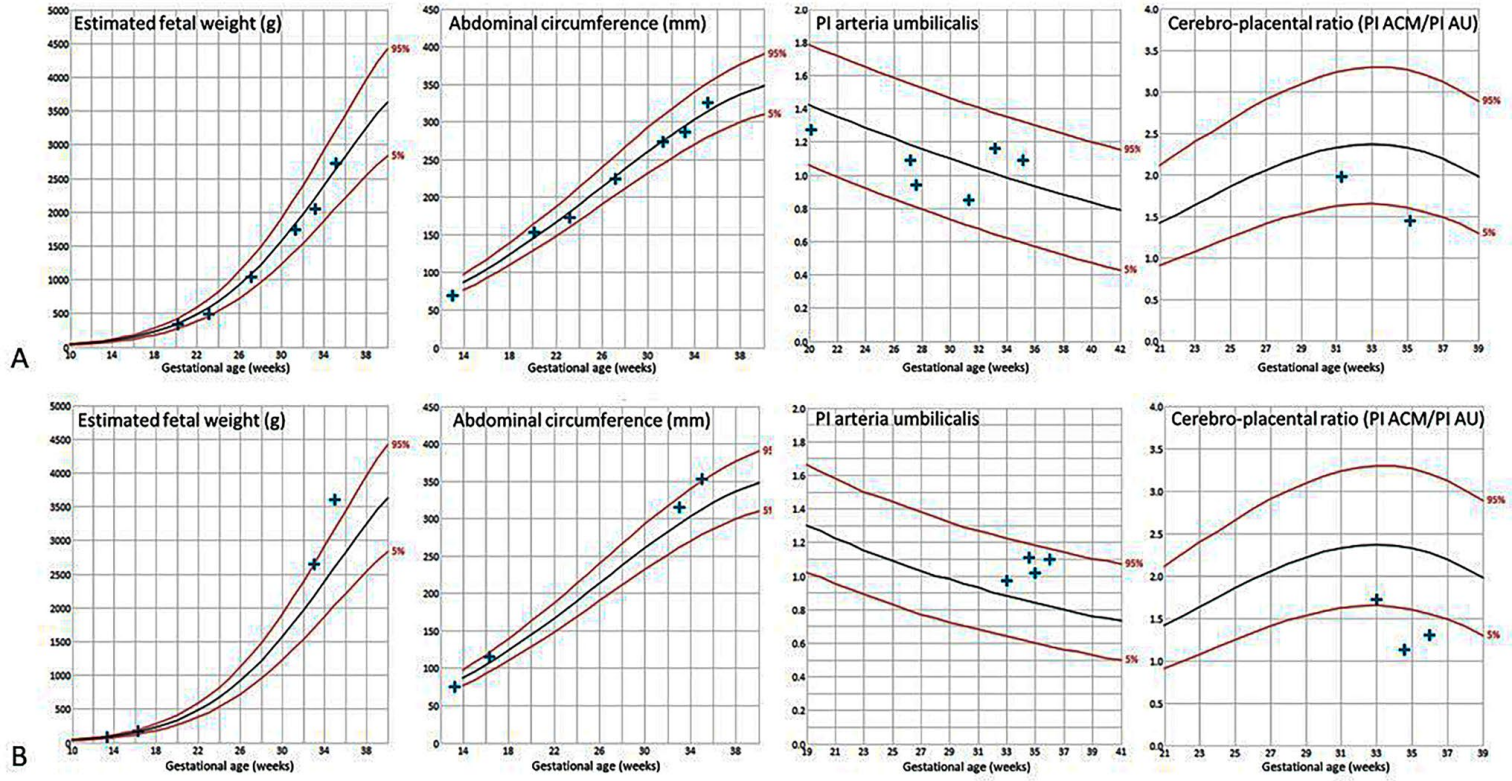
Longitudinal data of estimated fetal weight, abdominal circumference, pulsatility index of the umbilical artery and CPR during the reported pregnancy for case 2 in (**A**) and case 4 in (**B**)

At 6 weeks of gestation, the 19-year-old gravida I para 0 was presented to our maternity unit for the first time. Type 1 diabetes was diagnosed at the age of 2 years. The woman was treated with intensified insulin regime. Secondary diseases due to diabetes were not diagnosed yet. It was an unplanned pregnancy in poorly controlled type 1 diabetes. The HbA1c was 10.5% at the time of conformation of pregnancy. During the first trimester, we optimized the insulin regime and created an interdisciplinary team of obstetrics and diabetes specialist. Because of a small ventricular septum defect, an amniocentesis was performed, which revealed normal karyotype (46, XX). Repeated inpatient treatments were necessary to improve glucose control. Nevertheless, compliance was poor throughout pregnancy and was further worsened by the desolate social situation. Fetal growth was evaluated every second week, CTG and doppler measurements were performed weekly and revealed to be normal at any time. (Fig. 1A) Glucose control was insufficient and HbA1c was 9.4% at 34 weeks when the patient was constantly hospitalized and induction of labor was planned after completion of 37 weeks of gestation. CTG and Doppler remained normal and were controlled twice a week. (Fig. 1A) The day before transfer to the obstetric ward for induction of labor, fetal death had to be diagnosed during routine CTG. Labor was induced and after 24 h a female fetus with a birth weight of 3220 g (70% percentile) was born. The fetal autopsy showed cardiac malformations (ventricular septal defect) and signs of a diabetic fetopathy. The histological examination of the placenta described maternoplacental perfusion disorders with focal placental insults.

### 3rd Case

A 22-year-old gravida I para 0 (BMI 17 kg/m^2^) showed a pathologic fasting glucose of 6.7 mmol/l and HbA1c of 6.1% performed during routine pregnancy care in week 16. The patient declined further pregnancy care and no further blood glucose measurement or oral glucose tolerance test was performed. The next medical contact the women had in her pregnancy was at 32 weeks of gestation when stillbirth was diagnosed. At admission to our maternity unit the blood glucose level was 22.2 mmol/l without signs of ketoacidosis (pH 7.42) and the HbA1c was 13.5%. A therapy with short acting insulin was immediately started and labor was induced. A male fetus was born with a birth weight of 2080 g in the 32 weeks of gestation (> 97%). The metabolic condition of the mother remained stable. The diagnosis of Type 1 diabetes was confirmed by high levels of antibodies (auto-antibodies against glutamic acid decarboxylase 55.6 U/ml and tyrosine phosphatase IA-2 114 IU/l).

### 4th Case

From the beginning of her 3rd pregnancy, a 40 year-old gravida III para II (BMI 43 kg/m^2^) with a type 1 diabetes, was regularly counselled by her diabetes specialist as well as in our specialized obstetric unit. Type 1 diabetes was diagnosed 10 years after her first child was born. Blood glucose was managed using intensified insulin therapy. The medical history revealed obesity and arterial hypertension in addition to the type 1 diabetes but no other diabetes related diseases. With the beginning of the Corona pandemic the woman stopped attending most of her appointments and managed her blood glucose herself. With beginning of the 34 week of gestation her blood pressure raised and she developed symptoms of preeclampsia when she finally presented herself again at our outpatient unit. Her HbA1c level was increased to 7.3% and fetal biometry revealed an acceleration in the abdominal circumference and an estimated fetal weight above the 95th percentile. (Fig. 1B) Doppler parameters remained normal. (Fig. 1B) Inpatient treatment to treat preeclampsia symptoms and the poorly controlled diabetes was repeatedly refused by the patient. Finally she presented emergently with missing fetal movements and persistent vomiting since two days, when we could only diagnose fetal death in 36 weeks of gestation. After induction of labor, a macrosome male fetus was born with a birth weight of 4450 g (> 97% percentile).

## Discussion

Leading cause of intrauterine death during pregnancies is fetal growth restriction caused by placental dysfunction leading to fetal hypoxia and consecutive death. Chronic fetal hypoxia resulting from maternal and fetal hyperglycemia, indicated by the shown correlation between the concentration of erythropoietin in amniotic fluid and antenatal glycemic control, has been accounted for intrauterine death in diabetes [3]. Consistently, in an earlier study the authors recorded significantly higher HbA1c levels in the last trimester in 5 of 10 stillbirths in diabetic mothers [13]. In 2003, Lauenborg reported 25 cases of stillbirth in type 1 diabetic pregnancies and found in 9 of these cases no other cause of death than poor maternal glycemic control [14]. Additionally, the presence of microvascular damages and non-planned pregnancy has been reported to be associated with stillbirth in women with preexisting diabetes [15, 16].

We present four cases of stillbirth occurring in type 1 diabetes pregnancies. The presented cases did not follow a uniform pattern indicating that the same basic condition of type 1 diabetes causes intrauterine demise as a consequence of different underlying mechanisms. In three cases mothers were young and nulliparous, while one patient was in her forth decade and gave birth to two healthy children before. There was one case of planned pregnancy in contrast to three unplanned. HbA1c levels indicate poor glycemic control in two cases. There was one case with obesity and preexisting hypertension while there were normal BMI and lack of co-morbidities in all other cases. Duration of diabetes differed largely, with one patient being diagnosed at the age of two years and one during the reported pregnancy. Insulin therapy applied was by ICT in all three patients with known type 1 diabetes and all controlled their blood sugar by self-monitoring of blood glucose, none used sensor based glucose monitoring. All reported intrauterine deaths occurred during the third trimester.

Two of the dead born revealed to be macrosome with birth weight exceeded the 97th percentile according to gestational age. None was small for gestational age. No placenta was hypertrophic, in two cases placental weight revealed to be below the 3rd percentile. In these two cases the placental/fetal weight ratio was far above the 97th centile [12], although only one was accompanied by a big baby exceeding the 97th pctl. (Table 1) Doppler parameters revealed normal results for pulsatility indices of the umbilical and fetal cerebral arteries in the two cases where Doppler was performed. (Fig. 1) There was one case of ketoacidosis. Thus, the cases presented clearly demonstrate that the causes of IUFT in type 1 diabetes can be diverse.

The common feature in all four cases were absent or poor compliance and therapy adherence. Patient number 1’s non-compliance to medical advice lead to refuse of food intake and proper insulin treatment. In case two, despite the interdisciplinary care applied, the metabolic situation could not be stabilized within the target range. The patient’s lack of acceptance of the disease and the nonexistence of support from her social environment could not prevent the tragic outcome of the pregnancy despite inpatient observation. The patient in case three did completely refuse acceptance of the diagnosis and withdraw herself from medical control. In case four the patient was without professional diabetic assistance from week 20 to week 34, when the SARS-CoV-2 pandemic shut down public life. No external advice was obtained during that time.

Suboptimal maternal blood glucose levels, the presence of microvascular damages and poorly planned pregnancy [17] are described in the literature to be associated with increased risk of intrauterine death and can be considered to be part of the etiology of fetal demise in at least three of the four reported cases. Consequences of unplanned pregnancy and poor controlled diabetes are associated with high levels of HbA1c. Previous studies have shown that with each mmol/l the glucose level raises (above the requested 49 mmol/l or 6.7%) the relative odds for fetal or infant death raises by two to three %. The association between pre-conceptional HbA1c and the odds of fetal deaths is described as a J-shaped pattern. [18] However, in two of the four cases presented the HbA1c at the time of fetal demise was below 7% and thus are not in line with the literature to be simply explained by insufficient glycemic control.

Impaired placental function is a frequent causal pathway associated with stillbirth. [19] In the two cases presenting with a small placenta accompanied by big baby leading to detrimental fetal/placental ratio, placental dysfunction might be considered the cause of intrauterine death. However, in both cases established methods to monitor fetal wellbeing did fail to recognize fetal distress. Fetal growth did not slow down and Doppler parameters remained normal. Assessment of the cerebro-placental ratio (CPR), indicating redistribution of fetal blood flow to the brain, associated with fetal distress, was not introduced to clinical routine and its use is still restricted to the surveillance of growth retarded fetuses. However, CPR did not fall below one, which was considered as a threshold by Baschat and co-authors [20], but did fall below the 5th percentile [20] in the two cases Doppler values were available (Fig. 1).

A recent prospective cohort study of Paz on pregnant women with gestational diabetes could not show an association of adverse perinatal outcome and a CPR below the 10th pctl before delivery [21]. Gibbons and colleagues published in 2017 a big retrospective cohort study evaluating the association of CPR to perinatal outcome in diabetic pregnancies. Interestingly, they could only demonstrate an association of low CPR values with impaired perinatal outcome in the group with gestational diabetes, where low CPR was clearly associated with fetal growth restriction. In the two groups of pre-existing diabetes no association of low CPR to adverse perinatal outcome was shown. Remarkably, the authors could illustrate that women with pre-existing diabetes had lower CPR values compared to those with GDM, with the lowest values seen in the cohort with pre-existing type 1 diabetes. The low CPR values were accompanied by high values of the pulsatility index in the umbilical artery (PI AU) while the PI in the arteria cerebri media (PI ACM) did not change. This suggests, that the low CPR in this cases is mediated by increased resistance in placental perfusion possibly reflecting a diabetes associated end organ microvascular disease which involves the utero-placental circulation [22]. Microvascular diseases can be considered to be present in case two due to a history of badly controlled diabetes for more than 15 years and in case four due accompanying hypertension and preeclampsia. In these two, the microvascular disease most likely lead to placental insufficiency, as shown by placental hypotrophy. (Table 1) This underlines the importance of CPR assessment in diabetic pregnancies, especially in cases were the fetal growth does not fit with placental size.

As one cause of intrauterine death in diabetic pregnancies diabetic ketoacidosis (DKA) has been described [23]. DKA is characterizes by hyperglycemia in the absence of insulin leading to intracellular metabolism producing ketones. DKA occurs in 0.5–3% of all diabetic pregnancies (pregestational and gestational diabetes). Triggers for DKA are continuous vomiting, prolonged hypocaloric intake or even starvation, acute infections, poor control of blood sugar or poor compliance with treatment, insufficient self-management of diabetes therapy or glucocorticoid therapy. Pregnancy itself disposes to DKA due to the amount of contra insulinogenic hormones such as human placental lactogen in combination with the reduction of buffering capacity because of pregnancy induced respiratory alkalosis (compensatory renal loss of bicarbonate) and significant rise of insulin demand during the second trimester. As a result, DKA in pregnancy occurs even with low blood glucose levels and up to 30% of the DKAs during pregnancy can be euglycemic. DKA is associated with a high maternal morbidity and mortality [24, 25]. Fetal lethality depends on the severity of DKA and can reach up to 70% [26, 27]. Thus euglycemic DKA—as being described in the 1st case report—is an obstetric emergency and the outcome is dependent on imminent treatment [23].

The third case does not contain sufficient information to speculate on the reasons for fetal demise. Since we have no information on Doppler data placental insufficiency can neither be confirmed nor excluded. There were no ketoacidosis diagnosed at the time of intrauterine death and the fetal/placental ratio was within range. Leaving us with an unexplained intrauterine death of a macrosomic fetus and a corresponding HbA1c of 13.4% of the mother.

Notably, none of our cases was managed by sensor-based glucose monitoring. The features of sensor based glucose monitoring include the presence of complete glucose data even in patients with low compliance, like our patient in case two. Additionally, internet-based devices can be used to monitor glucose data online in patients where in person appointments are not possible, like in our case four were the patient was not seen by the diabetologist due to pandemic constrains. Additionally, disease insight might increase with more easily accessible glucose data by the patients themselves and management compliance can be increased by less burdened methods of glucose level determination.

## Conclusion

The main modifiable risk factors of stillbirth in diabetic pregnancies are maternal blood glucose levels including pre-conceptional HbA1c. Affected women should receive detailed pre-conceptional counselling. Appropriate disease acceptance improves coping and motivates to sufficient therapy compliance, improves blood glucose levels during pregnancy the prerequisites for a successful pregnancy outcome. Glucose control can be eased by implementing new techniques in diabetic management especially during pregnancy. However, late acute placental insufficiency in type 1 diabetes is a real threat, especially in high-risk women. Recommended monitoring intervals should be followed and fetal Doppler data should be obtained during the third trimester. The status of CPR and even more important the determination of the thresholds indicating fetal distress in normal and overgrown fetuses has to be investigated. In this context our presented cases emphasize the importance of close obstetric monitoring during the third trimester, including not only the monitoring of fetal growth but also Doppler parameters and possibly also the evaluation of placental size and growth in comparison to the fetus. Furthermore, pathomorphological examinations of placentas of all diabetic cases should be performed by researchers in order to define morphological changes caused by maternal hyperglycemia leading to placental insufficiency.

## Data Availability

All data generated or analysed during this study are included in this publication. Original data resources are available upon reasonable request from the corresponding author (tanja.groten@med.uni-jena.de).
